# Single Residue on the WPD-Loop Affects the pH Dependency of Catalysis in Protein Tyrosine Phosphatases

**DOI:** 10.1021/jacsau.1c00054

**Published:** 2021-04-23

**Authors:** Ruidan Shen, Rory M. Crean, Sean J. Johnson, Shina C. L. Kamerlin, Alvan C. Hengge

**Affiliations:** † Department of Chemistry and Biochemistry, Utah State University, Logan, Utah 84322-0300, United States; ‡ Science for Life Laboratory, Department of Chemistry − BMC, Uppsala University, Box 576, S-751 23 Uppsala, Sweden

**Keywords:** Protein tyrosine phosphatases, Enzyme kinetics, Protein dynamics, Enzyme catalysis, Point mutation, Loop dynamics, pH dependence, Molecular dynamics simulations

## Abstract

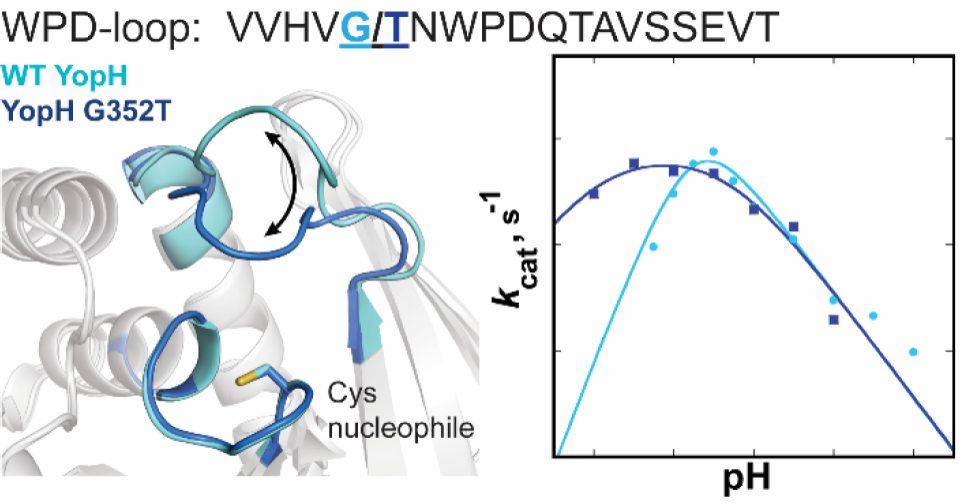

Catalysis by protein tyrosine phosphatases (PTPs) relies on the motion of a flexible protein loop (the WPD-loop) that carries a residue acting as a general acid/base catalyst during the PTP-catalyzed reaction. The orthogonal substitutions of a noncatalytic residue in the WPD-loops of YopH and PTP1B result in shifted pH-rate profiles from an altered kinetic p*K*_a_ of the nucleophilic cysteine. Compared to wild type, the G352T YopH variant has a broadened pH-rate profile, similar activity at optimal pH, but significantly higher activity at low pH. Changes in the corresponding PTP1B T177G variant are more modest and in the opposite direction, with a narrowed pH profile and less activity in the most acidic range. Crystal structures of the variants show no structural perturbations but suggest an increased preference for the WPD-loop-closed conformation. Computational analysis confirms a shift in loop conformational equilibrium in favor of the closed conformation, arising from a combination of increased stability of the closed state and destabilization of the loop-open state. Simulations identify the origins of this population shift, revealing differences in the flexibility of the WPD-loop and neighboring regions. Our results demonstrate that changes to the pH dependency of catalysis by PTPs can result from small changes in amino acid composition in their WPD-loops affecting only loop dynamics and conformational equilibrium. The perturbation of kinetic p*K*_a_ values of catalytic residues by nonchemical processes affords a means for nature to alter an enzyme’s pH dependency by a less disruptive path than altering electrostatic networks around catalytic residues themselves.

## Introduction

Enzymatic activity is usually pH-dependent, and this behavior is one of the standard parameters measured during in vitro kinetics studies of enzymes to identify catalytic residues and mechanisms. This factor has a more fundamental importance in biology, where an enzyme’s biological activity is affected by the pH of its microenvironment. This pH may or may not correspond to the optimum found in laboratory kinetics studies and can vary depending on the organism, within different compartments of the cell or cell type, and is different in healthy cells compared to the more acidic conditions within tumor microenvironments. In the protein tyrosine phosphatase (PTP) family, pH dependency is typically bell-shaped, with an optimum ranging from 4.5 for TK-PTP, found in the hyperthermophilic archaeon *Thermococcus kodakarensis*,^1^ to 7.5 for PTP gamma, a mammalian PTP implicated in human tumor suppression.^2^ The variations in pH optima among PTPs likely reflects optimization for their biological roles.

**Figure 1:**
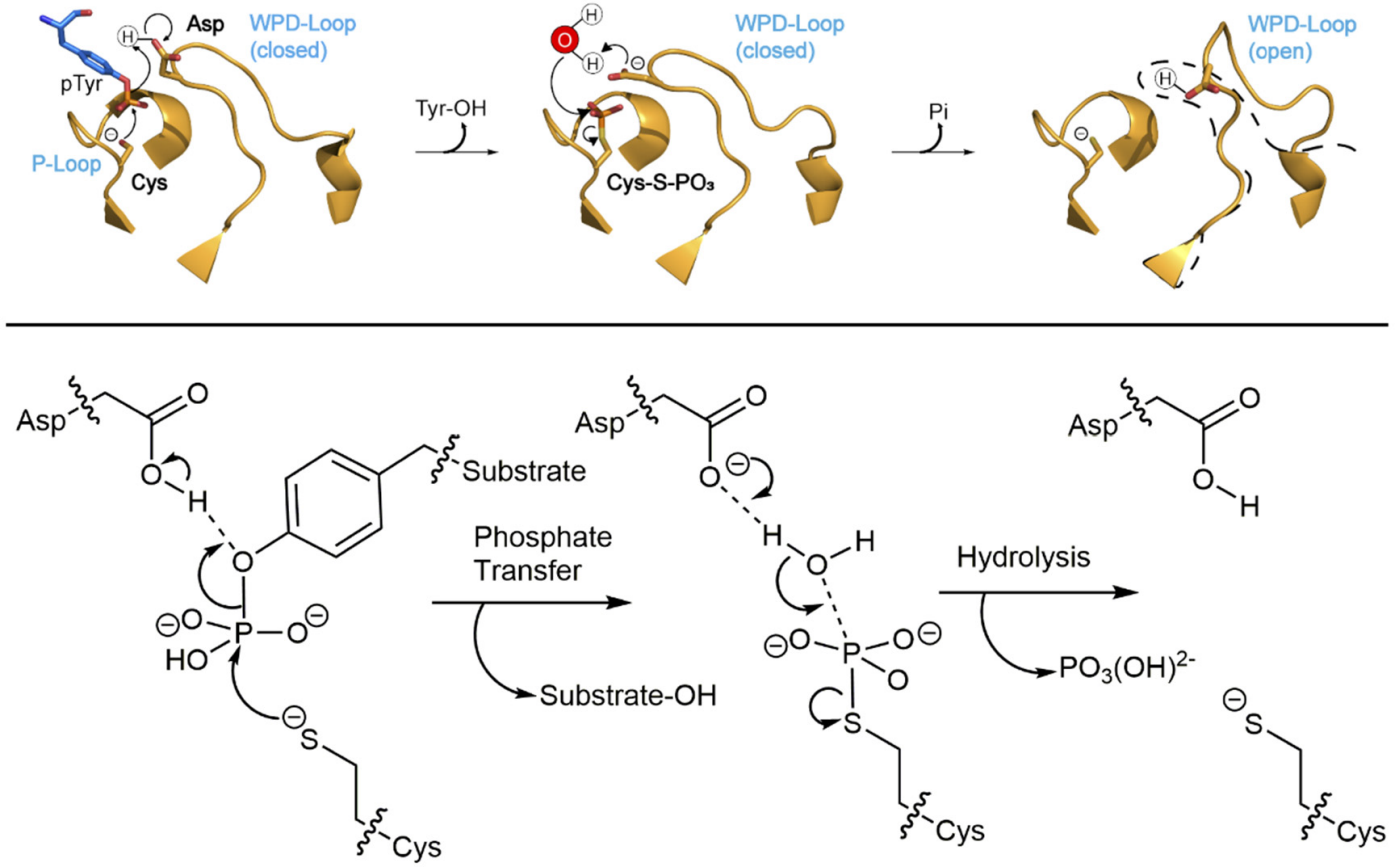
Mechanism of the PTP-catalyzed reaction, showing the mobile WPD-loop, the phosphate-binding P-loop, as well as the substrate, nucleophilic water molecule, and key catalytic side chains. Motion of the protein loop bearing the conserved general acid (i.e., the side chains of residues D356 in YopH and D181 in PTP1B) brings it into position to function as a general acid in the first step, where it protonates the leaving group during formation of a phosphocysteine intermediate. In the second step, the carboxylate form activates a nucleophilic water molecule.

The ubiquity of the PTP family in the biological kingdom reflects the fundamental role played by protein phosphorylation as a regulatory mechanism. The large family of diverse PTPs, together with protein tyrosine kinases, modulates the phosphorylation level of tyrosine residues in the cell. The classical PTPs hydrolyze only phosphotyrosine (pTyr) residues of polypeptide substrates, while the dual-specific subfamily members (DSPs) also dephosphorylate phosphoserine (pSer) and phosphothreonine (pThr) residues.^3−6^ All PTPs share the conserved signature motif HCX_5_R(S/T) forming the P-loop, which includes the nucleophilic cysteine and a conserved arginine, which, along with backbone amides, binds substrate and provides transition-state stabilization. PTPs follow a two-step catalytic mechanism with a cysteinyl–phosphate intermediate, shown in Figure 1. Many PTPs exhibit burst kinetics at pH ∼6, indicating that the first chemical step is rapid and the overall rate-determining step for *k*_cat_ is the hydrolysis of the intermediate under these conditions.^7−9^

In addition to the P-loop, PTPs share an aspartic acid that protonates the leaving group in the first chemical step and then, as a carboxylate, activates the nucleophilic water molecule in the second step. In the classical PTPs, this residue is found on a flexible loop of about a dozen residues of variable sequence with the exception of a highly conserved WPD sequence (Figure S1), leading to its designation as the WPD-loop. X-ray studies show a high degree of structural similarity in the P-loops and in the WPD-loops; the latter are usually, but not exclusively, found in the catalytically competent, closed position in PTP complexes with oxyanions in the active site^10−14^ and in a nonfunctional open position in ligand-free structures.^15,16^ Closure of the WPD-loop moves the conserved Asp residue about 9 Å into the position needed for catalysis (Figure S2). Despite structural similarities, catalytic rates vary over several orders of magnitude within the PTP family, even among the classic PTPs. In this context, NMR experiments have revealed a correlation between WPD-loop dynamics and substrate cleavage rate in YopH and PTP1B,^17^ and recent computational work similarly suggests that turnover rates by these enzymes are modulated by the conformational dynamics of the WPD-loops of these enzymes.^18^

Following from this, the active-site Cys nucleophile (Figure 1) must be deprotonated for catalysis, and the p*K*_a_ of this residue is significantly decreased from the typical solution value by the neighboring His residue, a conserved part of the signature P-loop motif. This His residue hydrogen bonds to the thiolate form of the Cys side chain, stabilizing the conjugate base form, and its mutation to Ala in YopH raises the Cys p*K*_a_ from 4.67 to 7.35.^7^ The Cys residue is found at the bottom of the active site in contrast to the other conserved catalytic residue, the general acid, which is in the solvent-exposed WPD-loop. This requirement for a deprotonated Cys and a protonated Asp gives rise to bell-shaped pH-rate profiles that are found for PTPs.^19^ However, despite the conservation of these two catalytic residues, the pH-rate profiles of some PTPs are notably broader than others, in addition to the aforementioned variation in the pH of optimal catalysis. For example, YopH has a narrower profile^20^ than PTP1B, and its maximal activity occurs over a narrower range.

However, the p*K*_a_ values reflected in a pH-rate profile extracted from a fit of the kinetic data to the appropriate equation reflecting the number of ionizable catalytic groups are often distorted from the thermodynamic or “true” p*K*_a_ of those groups.^21−23^ True p*K*_a_ values are reflected in the pH-rate profile only when a number of conditions are met. These include the condition of a single ionization state of the active site contributing to catalysis and when all prototropic equilibria (i.e., equilibria involving proton transfer processes) and other equilibria involving catalytic groups are fast with respect to the chemical steps. For example, substrate binding or dissociation that is not rapid relative to chemistry (referred to as sticky substrates) pushes the observed p*K*_a_ values outward from their true values.^24^ The two chemical steps of the PTP mechanism utilize different ionization states, so the rate-determining step will change with pH. Such a condition is known to affect the p*K*_a_ values observed in pH-rate profiles, one example being dihydrofolate reductase, where an active-site residue of p*K*_a_ 6.5 is observed at an apparent p*K*_a_ of 8.4.^25^ The correlation between protein motions and the rate of catalysis in the PTP family^17^ adds an additional complicating factor for this enzyme family. Specifically, contributions from WPD-loop dynamics to the chemical steps and how the rates of such motions vary with pH will affect the pH-rate profile and thus the apparent or “kinetic” p*K*_a_ values.

**Scheme 1:**
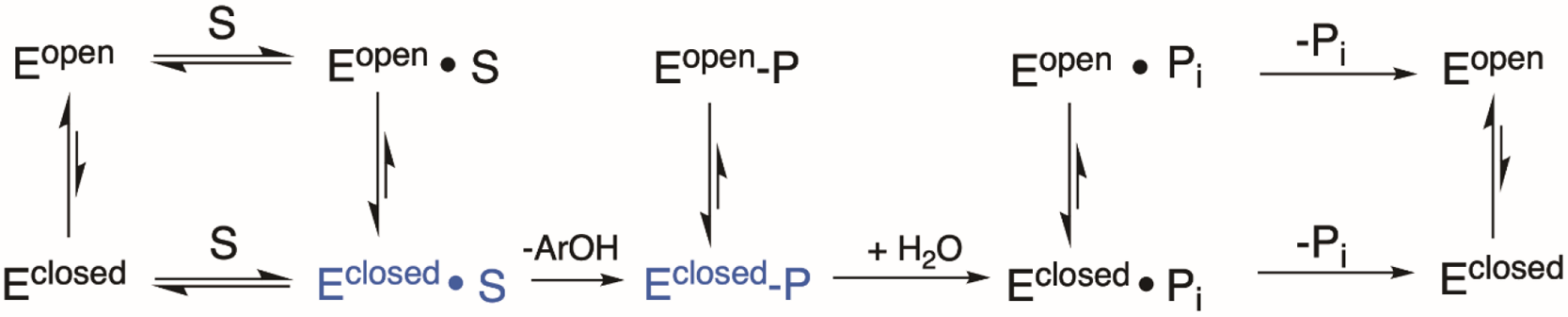
Kinetic Scheme for Conversion of Substrate to Product and Release, Including Equilibria of the WPD-Loop Prototropic equilibria of the catalytic residues have been omitted. The catalytically functional species are shown in blue. Substrate can bind and product can be released from either loop-open or -closed forms, but the chemical steps can only occur from loop-closed conformations. Any factor that alters equilibria between open and closed forms may affect the extent to which the first or second step is rate-limiting at a given pH, affecting p*K*_a_ values observed in a pH-rate profile.

Scheme 1 shows a simplified kinetic scheme for PTP catalysis including WPD-loop-open and -closed conformations. Because the loop moves the general acid into the active site from the side, rather than over the top of the active site, the substrate can bind to either the closed or open forms of the WPD-loop. However, substrate binding shifts the equilibrium from favoring the loop-open form to favoring the closed form, and only the loop-closed form can undergo catalysis in either of the chemical steps, highlighted in blue in Scheme 1. Loop equilibria affect the relative populations of the catalytically competent Michaelis complex and the phosphoenzyme intermediate. The pH will likely affect this equilibrium, as well as directly control the degree to which the first or second chemical step is rate-limiting. Both of these factors, in turn, perturb the apparent p*K*_a_ values obtained from kinetic measurements from their thermodynamic values.

The kinetic p*K*_a_ values, manifested in the catalytic reaction as a function of pH and which can differ from the thermodynamic ones,^21−23^ describe the biological activity of an enzyme in a particular environment. The divergence between kinetic p*K*_a_ values and the true thermodynamic p*K*_a_ values of catalytic groups provides a means for nature to tune the pH dependency of enzymatic activity that complements the well-established means by which proteins can alter the inherent thermodynamic p*K*_a_ values of catalytic groups by modifying their electrostatic environment.^26^ In the PTP family, in particular, alterations in residues that affect protein motions that are connected to catalysis, particularly those of the WPD-loop, have the potential to alter the pH dependency of the reaction without changes to interactions to either the nucleophilic Cys residue at the bottom of the active site or the conserved aspartic acid on the mobile WPD-loop (Figure 1).

As proof of this concept, we have identified a mutation to a noncatalytic residue on the WPD-loop of YopH that significantly increases its activity at low pH. The resulting broadening of the pH dependency of catalysis of the G352T variant bestows more than an order of magnitude faster turnover at pH 4, with no loss of activity at its pH optimum compared to the wild type (WT). Structural characterizations and computational modeling show how this mutation shifts the equilibrium between the WPD-loop-open and -closed states. The kinetic p*K*_a_ of the Cys residue responsible for the lower limb of the pH-rate profile is lowered in G352T by nearly a full unit, while titration of the active-site Cys residue shows only a small change in its thermodynamic p*K*_a_. Interestingly, the corresponding T177G variant of PTP1B shows a more modest effect and in the opposite direction. The documented correlation between WPD-loop dynamics and catalysis in YopH and PTP1B^17,18^ provides a rationale for the altered pH dependencies of these variants.

## Materials and Methods

### Chemicals

Dithiothreitol (DTT) and ampicillin (AMP) were purchased from GoldBio. Restriction enzymes were purchased from Integrated DNA Technologies. Protease-inhibitor tablets were purchased from Sigma-Aldrich. All other buffers and reagents were purchased from Sigma-Aldrich or Fisher. The substrates *p*-nitrophenyl phosphate (*p*NPP)^27^ and *p*-nitrophenyl phosphorothioate (*p*NPPS)^28^ were synthesized using published methods. Crystallography screens, trays, and coverslips were purchased from Hampton Research.

### Mutagenesis and Expression

The plasmid pEt-19b encoding wild-type human protein PTP1B was provided by Dr. N. K. Tonks, and the plasmid pEt-43.1b (+) encoding wild-type *Yersinia* PTP was provided by Dr. Z.-Y. Zhang.

PTP1B T177G was made by substituting residue T177 of wild-type PTP1B via the Q5-SDM kit (New England Biolabs) with G from the YopH WPD-loop in the corresponding region (Table S1). YopH G352T was made by the same method, replacing residue G352 of wild-type YopH with T from PTP1B in the corresponding region. Both point mutants were created using polymerase chain reaction (PCR) with primers encoding residues before the mutation and the target mutation itself. The mutant DNA was then cleaved using the restriction enzyme DpnI and ligated into either the pEt-19b or pEt-43.1b (+) vector using T4 ligase. The primers used are listed in Table S2.

The DNA was transformed into BL21-DE3 cells and grown overnight at 37 °C on an LB culture plate containing 10% ampicillin. One colony was selected and placed into 10 mL of SOC media containing 10% ampicillin and grown overnight. The following morning, 1 L of LB media containing 10% ampicillin was inoculated with the 10 mL of overnight growth and shaken at 170 rpm at 37 °C until the OD_600_ reached 0.6–0.8. After the optimal OD was reached, the 1 L growth was induced by 0.1 mM isopropyl β-d-thiogalactoside (IPTG) and shaken at 170 rpm at room temperature overnight. The cells were harvested by centrifugation at 12,000*g* for 30 min at 4 °C and stored at −80 °C.

### Protein Purification

Both mutants were expressed and purified as for the wild-type parent PTPs.^19,29^ PTP1B T177G cells were thawed on ice and resuspended in 10× their equivalent volume of a lysis buffer, consisting of 50 mM imidazole pH 7.5, 1 mM EDTA, 3 mM DTT, and 10% glycerol with 0.5 mg/mL aprotinin, 0.7 mg/mL pepstatin, and 0.5 mg/mL leupeptin. YopH cells were thawed and resuspended in 100 mM acetate pH 5.7, 100 mM NaCl, 1 mM EDTA, 3 mM DTT, and 10% glycerol with the same protease inhibitors. The cells were lysed by sonication at 60% power for 10 pulses and then mixed on ice for 1 min and repeated 4–6 times until completely lysed. The cell lysate was centrifuged at 4 °C at 29000*g* for 30 min. The supernatant was filtered with a 0.45 μm syringe filter.

The filtrate was then purified via a 5 mL HiTrap Q HP column attached above a 5 mL HiTrap SP HP column using an FPLC filtration system. Both columns were equilibrated with lysis buffer. The cell lysate was loaded onto the columns at 1.5 mL/min; the HiTrap Q HP column was removed after loading, and the HiTrap SP HP column was washed with lysis buffer until the absorbance at 280 nm baselined. Elution for PTP1B T177G was processed using a 100% gradient with elution buffer containing 500 mM NaCl, 50 mM imidazole pH 7.5, 1 mM EDTA, 3 mM DTT, and 10% glycerol, and YopH with 100 mM acetate pH 5.7, 500 mM NaCl, 1 mM EDTA, 3 mM DTT, and 10% glycerol. Eluted fractions exhibiting absorbance at 280 nm were collected and tested with *p*NPP for phosphatase activity. Fractions that showed activity were assayed for purity on a 15% SDS-PAGE gel.

The active fractions were pooled (ranging from 30 to 40 mL) and concentrated to <12 mL, loaded onto a pre-equilibrated HiLoad 26/60 Superdex 200 prepgrade column (GE), and purified using 10 mM Tris buffer pH 7.5, with 25 mM NaCl, 0.2 mM EDTA, and 3 mM DTT for PTP1B T177G, and 100 mM acetate pH 5.7, 100 mM NaCl, 1 mM EDTA, 3 mM DTT for YopH G352T. Fractions were assayed with *p*NPP for activity and purity on a 15% SDS-PAGE gel. Pure protein was concentrated to 10–35 mg/mL and either kept on ice to immediately set up crystal trays or diluted with 10% glycerol and frozen with liquid nitrogen and stored at −80 °C in aliquots.

### X-ray Crystallography

Crystals for PTP1B T177G were grown by hanging drop vapor diffusion using 15 mg/mL protein and a well solution of 0.1 M Tris hydrochloride pH 7.5–8.5, 0.2 M magnesium acetate tetrahydrate, and 20–25% PEG 8000 at a 2:2:0.5 protein/well/20% benzamidine hydrochloride drop ratio. The vanadate-bound structures were obtained by adding 5 mM sodium metavanadate (Na_3_VO_4_) to the protein for cocrystallization. Crystals were transferred to a cryo-protectant solution containing mother liquor, 20% benzamidine hydrochloride, and 50% sucrose before flash freezing in liquid nitrogen.

Crystals for YopH G352T were grown by hanging drop vapor diffusion using 2 mg/mL protein and a well solution of 0.1 M HEPES pH 7.5 and 15–34% PEG 3350 at a 2:2 protein/well drop ratio; 0.5 μL seeding stock was then added to the drop. The microcrystals were generated by crushing 1 μL of PTP1B T177G crystals in the well solution and diluted to 1:10^8^ ratio. The vanadate-bound structures were obtained by adding 5 mM sodium metavanadate (Na_3_VO_4_) to the protein for cocrystallization. Crystals were transferred to a cryo-protectant solution containing mother liquor with 5 mM sodium metavanadate and 20% glycerol before being flash frozen in liquid nitrogen.

Diffraction data were collected on Stanford Radiation Lightsource (SSRL) beamline 9-2 (Table 1). Molecular replacement was performed with Phaser-MR^30^ as implemented in Phenix^31^ using WT PTP1B (PDB ID: 3I80(10)) and YopH W354Y (PDB ID: 4YAA(32)) as search models for PTP1B T177G and YopH G352T, respectively. Phenix.refine^30^ was used for refinement. Model building was performed using Coot.^33^ All figures of the enzyme structures and structural alignments therein were made using Pymol (The PyMOL Molecular Graphics System, version 1.2r3pre, Schrödinger, LLC).

**Table 1:** Data Collection and Refinement Statistics

	PTP1B T177G (ligand-free)	PTP1B T177G (VO_4_)	YopH G352T (ligand-free)	YopH G352T (VO_4_)
PBD ID	7L0C	7L0H	7L0I	7L0M
**Data Collection**				
source	SSRL 9-2	SSRL 9-2	SSRL 9-2	SSRL 9-2
space group	*P*3_1_21	*P*3_1_21	*P*2_1_2_1_2_1_	*P*2_1_2_1_2_1_
Cell dimensions				
*a*, *b*, *c* (Å)	88.506, 88.506, 104.432	88.471, 88.471, 104.979	49.183, 56.351, 98.513	49.231, 56.091, 98.302
α, β, γ (deg)	90, 90, 120	90, 90, 120	90, 90, 90	90, 90, 90
resolution (Å)	43.2–1.80	43.3–2.10	49.2–2.02	49.2–2.00
	(1.86–1.80)	(2.18–2.10)	(2.10–2.02)	(2.10–2.00)
CC_1/2_	0.699	0.632	0.467	0.796
*I*/σ*I*	31.5 (0.9)	29.8 (1.3)	22.2 (14.5)	88.1 (8.6)
completeness (%)	99.9 (100.0)	100.0 (100.0)	94.3 (79.6)	95.7 (81.0)
redundancy	18.9 (14.8)	18.6 (14.1)	5.5 (3.0)	7.7 (5.0)
no. reflections	44,193 (4324)	28,286 (2773)	17,623 (1437)	18,269 (1520)
**Refinement**				
*R*_work_/*R*_free_	0.163/0.175	0.178/0.208	0.202/0.253	0.178/0.226
no. atoms				
protein	2390	2425	2117	2153
ligand/ion	0	5	15	5
water	293	147	148	145
β-factors				
protein	28.9	49.5	34.9	32.3
ligand/ion		39.4	42.0	28.2
solvent	39.8	49.9	35.8	35.7
rms deviations				
bond lengths (Å)	0.007	0.009	0.007	0.008
bond angles (deg)	0.97	1.17	0.93	1.11
Ramachandran				
favored (%)	97.3	97.6	96.4	96.4
allowed (%)	2.4	2.0	3.2	3.2
outliers (%)	0.3	0.3	0.4	0.4

### Steady-State Kinetics

Steady-state kinetic parameters were measured at 25 °C. Concentrated protein aliquots were thawed on ice and diluted with a buffer base mix (BBM) containing 50 mM sodium acetate, 100 mM Tris, and 100 mM bis-Tris from pH 4.0 to pH 7.5. This buffer system maintains constant ionic strength throughout the pH range examined. A 50 mM solution of the dicyclohexylammonium salt of *p*NPP was prepared in the buffer base mix. The reactions were run on a 96-well plate using diluted enzyme protein concentrations from 0.0084 to 0.0106 μM (PTP1B T177G) or 0.0004–0.0007 μM (YopH G352T) and substrate concentrations from 0.76 to 22.73 mM. Reactions were allowed to proceed for 2 min for PTP1B T177G and 3 min for YopH G352T. The reactions were quenched using 50 μL of 5 M NaOH, and the amount of the product *p*-nitrophenol was assayed from the absorption at 400 nm using the molar extinction coefficient of 18,300 M^–1^ cm^–1^. Reaction blanks were made using identical conditions replacing the enzyme with buffer to correct for non-enzymatic hydrolysis of the substrate. The amount of product released and elapsed time were used to calculate the initial rates. The data were fitted to the Michaelis–Menten equation to obtain steady-state kinetic parameters. Kinetic data were obtained on both mutants as a function of pH to obtain pH-rate profiles. The bell-shaped pH-rate profiles were fitted to eq 1, the standard equation relating the dependence of the observed *k*_cat_ to the maximal, or limiting, value as a function of pH, where catalysis is dependent on two ionizable residues, one protonated and the other deprotonated.^34^
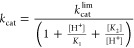
1

### Active-Site Cysteine Thiol Titrations

Enzyme stock was diluted in buffer base mix solution (100 mM sodium acetate, 50 mM bis-Tris, 50 Mm Tris-HCl) at various pH values with 1–10 mM iodoacetate. Reaction blank was made with the same reagents omitting the iodoacetate. Enzyme was incubated with iodoacetate and an aliquot was removed at 5–10 min intervals to assay for activity with 3.8 mM *p*NPP. Phosphatase activity assays were allowed to proceed for 180 s in BBM at pH 5.0 and quenched with 5 M NaOH. Formation of *p*-nitrophenol product was assayed as described above. Experiments were done at 0.5 pH unit intervals from pH 4.0 to pH 7.0.

At each pH, residual activities as a function of time were plotted to obtain *k*_obs_ (min^–1^) for the inactivation rate constant at each iodoacetate concentration. These *k*_obs_ values were plotted against iodoacetate concentrations to obtain the second-order rate constant *k*_2_ (M^–1^ min^–1^) as a function of pH. The pH dependency of iodoacetate inactivation was obtained by plotting *k*_2_ as a function of pH (Figure S4) and fitted to eq 2 to obtain the thermodynamic p*K*_a_ value for the active-site cysteine thiol. The same methodology has been used to obtain the cystine p*K*_a_ values in YopH^7^ and VHR.^35^
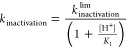
2

### Molecular Dynamics Simulations and Analysis

Molecular dynamics (MD) simulations were performed using the AMBER 18^36^ simulation package with the ff14SB^37^ force field and TIP3P^38^ water model to describe protein atoms and water molecules, respectively. Simulations of both WT PTPs and their corresponding point mutants were initiated where possible from either the corresponding X-ray crystal structure obtained from this study or structures used in previous studies (see Table S3 for all structures used).^14,39,40^ Simulations were performed on the ligand-free forms of each enzyme variant. For the open WPD-loop conformations of T177G and G352T point mutants for which no crystal structures exist, the WT crystal structures (with substitutions made using the Dunbrack rotamer library^41^ as implemented in PyMOL, selecting the side chain rotamer that minimizes steric clashes with the surroundings) were used as the starting structure for the MD simulations. MolProbity^42^ was used to identify any necessary Asn, Gln, and His rotamer changes, whereas PROPKA v.3.1^43^ was used to assign protonation states to simulate at an effective pH of 5 for all enzymes (see Table S4 for all residues assignments), with care taken to be consistent for simulations of the same PTP. Structures were solvated in an octahedral water box (keeping all crystallographic waters) such that all solute atoms were at least 10 Å away from the box boundaries, and Na^+^ or Cl^–^ ions were added as necessary to ensure system neutrality.

We followed a standard minimization, heating, and equilibration protocol (described in full in the Supporting Information) to prepare each structure for production of MD simulations in the NPT ensemble (300 K, 1 atm). For each system, 10 replicas of 100 ns long MD simulations were performed starting from both the closed and open WPD-loop conformations. MD simulations were run with a 2 fs time step (with the SHAKE^44^ algorithm applied) and an 8 Å direct space nonbonded cutoff, with long-range electrostatics evaluated using the particle-mesh Ewald^45^ method. Temperature and pressure were regulated using Langevin temperature control (collision frequency of 1 ps^–1^) and a Berendsen barostat (pressure relaxation time of 1 ps). Simulation analysis was performed using a combination of CPPTRAJ^46^ and the R software package^47^ (for statistical tests). Residues 176–190 and 351–565 of the WPD-loops of PTP1B and YopH, respectively, were used for the C_α_ root mean square deviation (RMSD) calculations. Hydrogen bonds were defined as being present if the donor–acceptor distance was ≤3.5 Å and if the donor–hydrogen–acceptor angle was 180 ± 45°.

## Results and Discussion

### X-ray Crystal Structures

**Figure 2:**
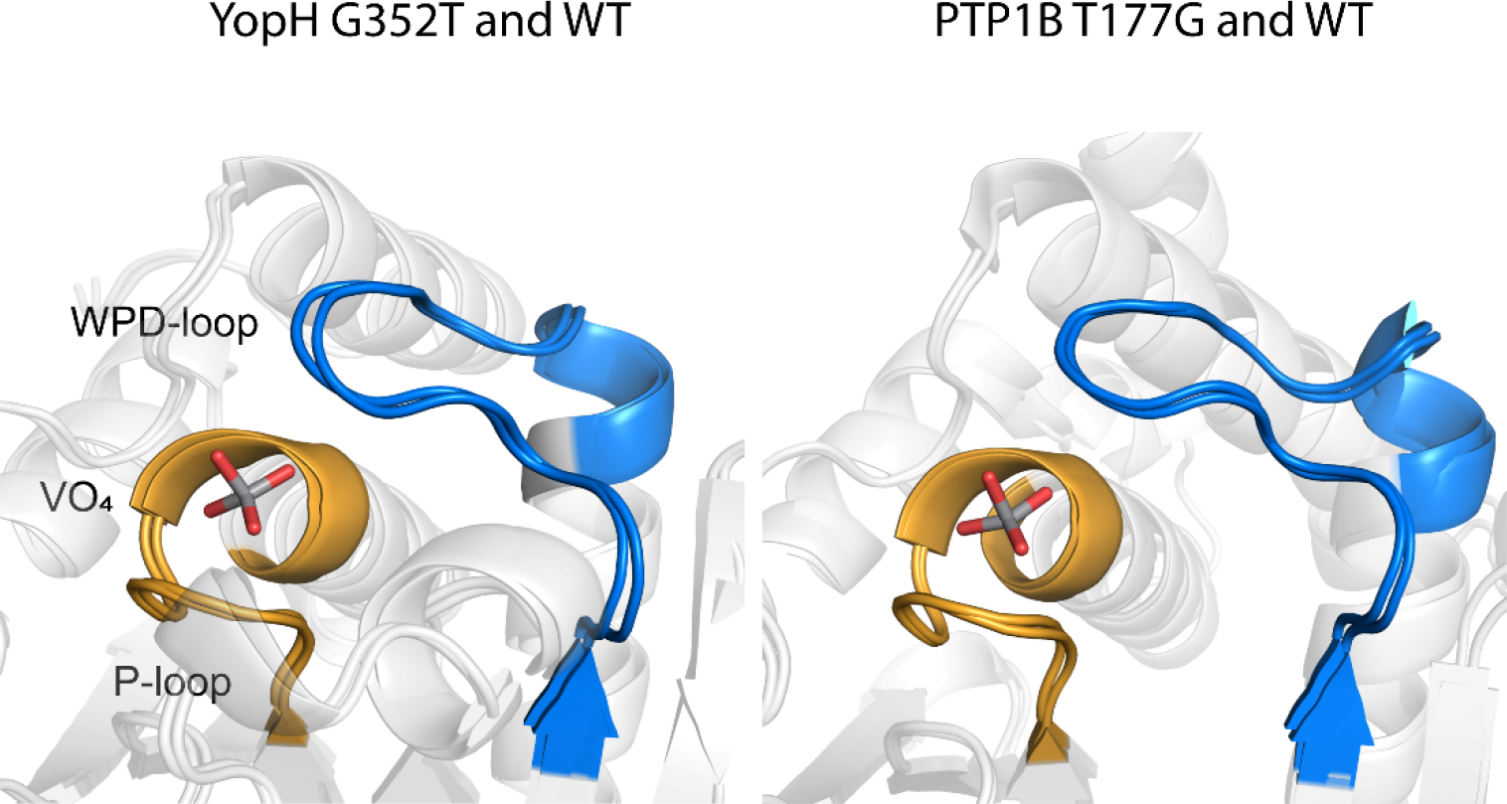
Comparison of active-site structures of ligand-bound mutant PTPs match well with those for their wild-type counterparts. The remainder of the scaffold is virtually identical between the liganded and ligand-free forms of each enzyme (backbone RMSD of 0.461 and 0.233 Å for PTP1B and YopH, respectively). Left: Vanadate-bound WT YopH (PDB ID: 2I42(40)) and vanadate-bound G352T YopH (this work). Right: vanadate-bound WT PTP1B (PDB ID: 3I80(10)) and vanadate-bound T177G PTP1B (this work). Ligand-bound WPD-loops are colored in blue, and ligand-bound P-loops are colored in yellow.

Structures were obtained of the PTP1B T177G and YopH G352T ligand-free enzymes and in complex with vanadate. Comparison of the variant structures with those of the native enzymes show no significant backbone perturbations in the WPD-loop regions or elsewhere (Figure 2).

**Figure 3:**
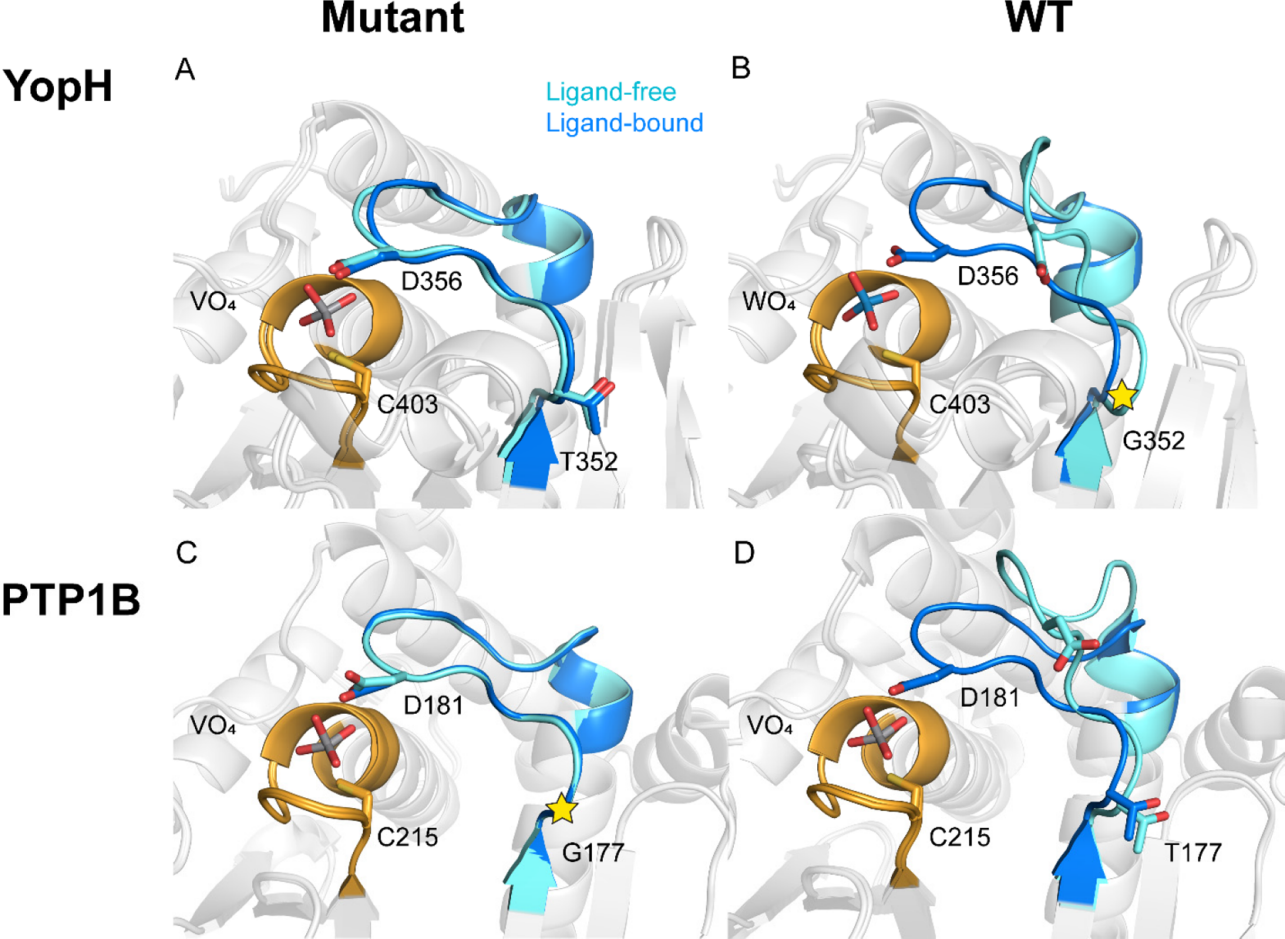
Active-site structures of mutant PTPs crystallize in the closed WPD-loop conformation in both ligand-free and ligand-bound conditions, indicating a shifted loop equilibrium. (A) Ligand-free and vanadate-bound G352T YopH (this work). (B) Ligand-free (loop open) (PDB ID: 1YPT(14)) and tungstate-bound (PDB ID: 1YTW(12)) (loop closed) complexes of WT YopH. (C) Ligand-free and vanadate-bound PTP1B T177G (this work). (D) Ligand-free WT PTP1B (PDB ID: 2CM2(15)) and vanadate-bound WT PTP1B (PDB ID: 3I80(10)). The WPD-loops in ligand-free enzymes are colored in cyan, ligand-bound WPD-loops are colored in blue, and P-loops are colored in yellow. Vanadate ions are only present in ligand-bound forms. The positions of G352 in YopH and G177 in PTP1B are marked by the star symbols.

In both ligand-free structures, the WPD-loops are observed in the closed position (Figure 3). This is in contrast to the typical observation of this loop in the open, noncatalytic position in crystal structures of these and other PTPs lacking a bound oxyanion. There has been no X-ray structure reported of ligand-free WT YopH with its loop in the closed conformation. There is an example of such a structure of PTP1B;^48^ however, this structure (PDB ID: 1SUG(48)) was subsequently re-refined and found to contain electron densities indicating the presence of both open and closed WPD-loop states.^39^ In contrast, we do not observe any electron density for the open state in the crystal structure of PTP1B T177G. Particularly, for the YopH variant, the closed conformation found suggests the G352T mutation has shifted the equilibrium of the ligand-free enzyme to favor the closed conformation.

The G352T substitution in YopH introduces a new 3.3 Å hydrogen bond between the side chain of T352 and the backbone carbonyl of M328 (Figure S3) in both the ligand-free and vanadate-bound structures, stabilizing the loop-closed conformation. If this hydrogen bond is only present, or is tighter, in the loop-closed conformation, it would rationalize the presence of this conformation in the ligand-free structure in contrast to the native enzyme. We were unable to obtain a crystal structure of a loop-open conformation of the mutant, and interactions of T352 in that conformation are unknown.

**Figure 4:**
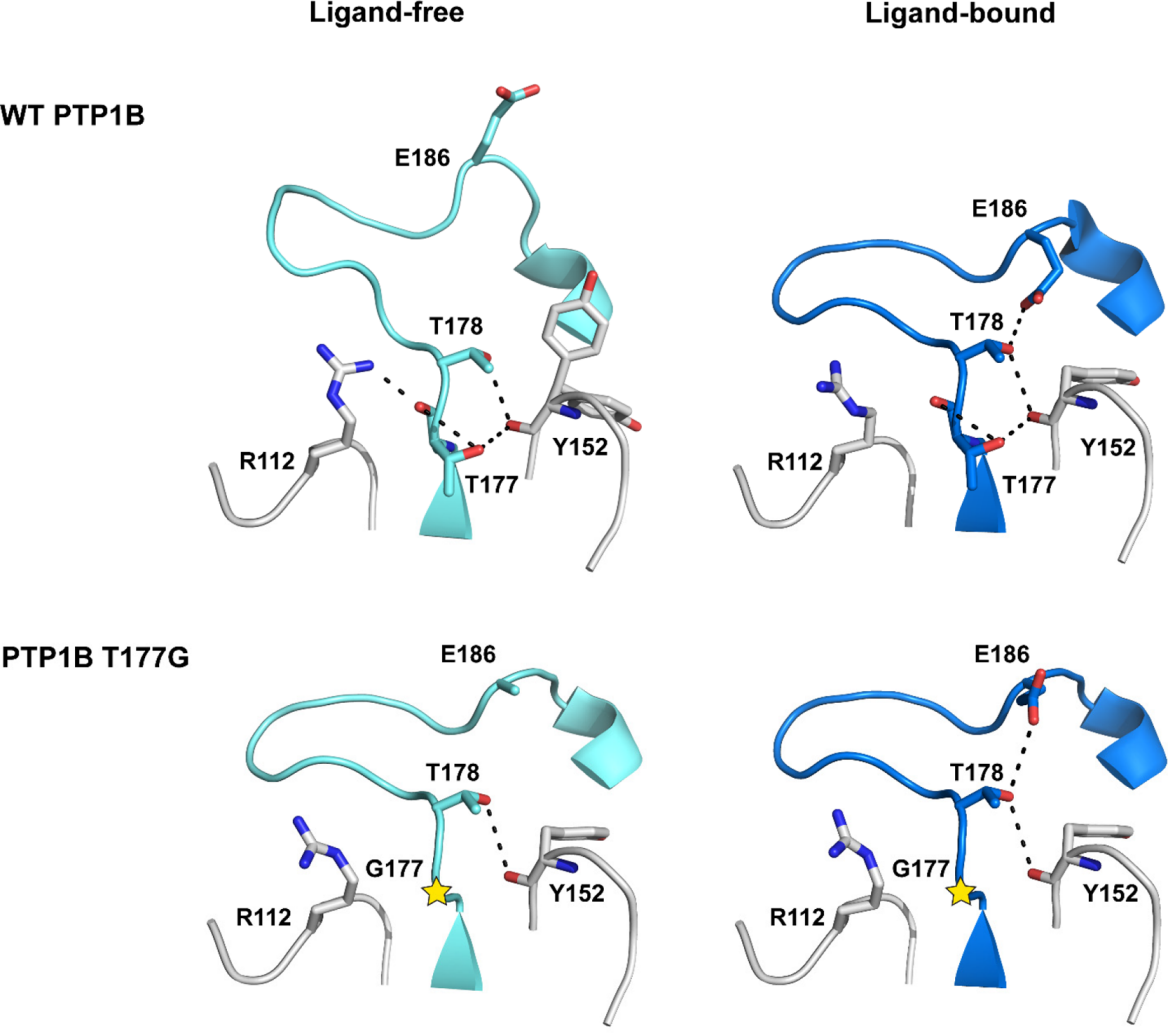
Interaction between R112 and T177 in loop open form of WT PTP1B is lost in the loop-closed form and in PTP1B T177G. The guanidinium side chain forms a hydrogen bond in the loop-open form, while the side chain rotates away from the WPD-loop in the closed form. The side chain conformation of E186 is uncertain in the crystal structure of ligand-free PTP1B T177G (this work). Vanadate-bound WT PTP1B (PDB ID: 3I80(10)) and ligand-free WT PTP1B (PDB ID: 2CM2(15)) are previously reported structures. The positions of G177 in PTP1B structures are marked by the star symbols.

For the PTP1B variant, crystal structures do not provide a clear rationale for how the T177G mutation might affect loop equilibrium. Residue T177 is involved in a network of hydrogen bonds in native PTP1B. In the WPD-loop open conformation, the backbone amide of T177 hydrogen bonds both with the side chain of R112 and with the threonine hydroxyl of T177. In the loop-closed conformation, the interaction with R122 is lost and the internal hydrogen bond with the hydroxyl group has lengthened from 3.3 to 3.5 Å (Figure 4). The loss of the R122 interaction results from the rotation of the guanidinium side chain and the resulting new hydrogen bonds to the bound substrate or oxyanion inhibitors in the loop-closed conformation. As shown in Figure 4, a comparison of ligand-free native PTP1B in WPD-loop-open and -closed states shows that the primary difference involving T177 is loss of the hydrogen bond between the backbone carbonyl of T177 and the guanidinium side chain of R122 in the closed state and a slight tightening of the two hydrogen bonds of the side chain hydroxyl.

In native PTP1B, the backbone amide carbonyl of Y152 is within the hydrogen bonding distance of the hydroxyl groups of T177 and T178 in both WPD-loop conformations. In this way, T177 and Y152 act as anchors to the interaction between T178 and E186 (Figure 4). In the loop-open conformation, the carboxylic acid group of E186 changes its direction from pointing toward the N-terminal of the loop to away from T177; therefore, the polar interaction between these residues is not observed in the opened loop. In the mutant PTP1B structure, most of the interactions that stabilize the closed conformation remain intact; however, the polar interaction between the side chain of T177 and the backbone of Y152 is lost. In addition to the loss of this interaction between T177 and Y152, no other obvious changes in the X-ray crystal structures indicate alterations of the WPD-loop equilibria. This is consistent with the observation that the T177 side chain is part of a network of noncovalent interactions that are likely responsible for stabilizing the open conformation of the WPD-loop, and therefore substitutions at position 177 would be expected to disrupt this interaction network, causing a population shift toward a closed conformation of the loop.^49^ We note that the observation of a closed loop in both ligand-free and bound forms does not infer that the WPD-loop has been rigidified and is no longer mobile. That is, retention of loop motion is supported by NMR experiments on the related PTP1B T177A variant, which showed that the loop still samples both closed and open conformations but with a shift in population to favor the closed conformation.^49^

### Kinetics

**Figure 5:**
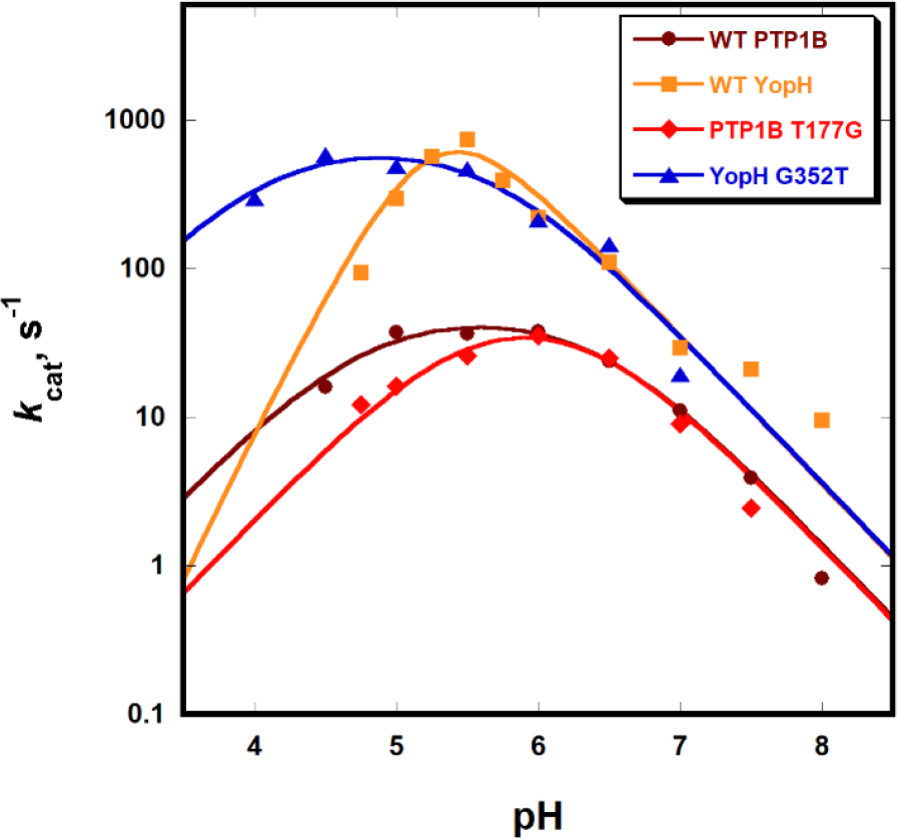
YopH G352T variant shows higher enzymatic activity at low pH than WT due to altered kinetic p*K*_a_ values resulting in a broader pH-rate dependency. The orthogonal T177G PTP1B mutant shows more modest effects in the opposite direction, a slight narrowing of the profile, and lower activity at low pH compared to that with WT.

Figure 5 shows the pH dependency of the turnover number with the substrate *p*NPP for the native enzymes and variants. A modest change to the profile for PTP1B is observed in the T177G variant. The basic limb of the profile and the maximal turnover number are unaltered, while the acidic limb is shifted somewhat to higher pH, with the net result that the T177G variant below pH 6 is a slower enzyme than native PTP1B. An opposite and more dramatic effect occurs in YopH. The WT YopH kinetic p*K*_a_ values (Table 2) are each ∼0.4 units higher than those previously reported at a higher temperature and different buffer system.^20^ The difference between them is nearly identical to the previous report, and this smaller difference in YopH relative to PTP1B gives rise to its narrower pH profile. Its G352T variant shows a much broader pH-rate profile than WT, with a kinetic p*K*_a_ of the nucleophilic Cys residue lower by about a full unit (Table 2). The native YopH enzyme has a pH optimum lower than that of PTP1B, 5.5 versus 6, and in the variant, it is reduced to 4.8. The variant and native YopH have very similar activities at their optimal pH, but the variant has significantly more activity at pH <5, by approximately an order of magnitude at pH 4.5.

**Table 2:** Kinetic p*K*_a_ Values for Wild-Type Enzymes and Mutants Obtained from Fits of the pH-Rate Data^a^

enzyme	p*K*_a1_	p*K*_a2_
WT PTP1B	5.08 ± 0.20	6.44 ± 0.26
PTP1B T177G	5.44 ± 0.37	6.37 ± 0.28
WT YopH	5.02 ± 0.27	5.58 ± 0.34
YopH G352T	4.06 ± 0.23	5.70 ± 0.24

a The p*K*_a1_ value arises from the cysteine nucleophile and p*K*_a2_ from the aspartic acid.

The fits of pH-rate data to eq 1 show negligible changes to the p*K*_a_ of the aspartic acid, reflected in the basic limb in the variants, but clear changes are present in the acidic limb, as reflected in the kinetic p*K*_a1_ values in Table 2. The T177G mutation in PTP1B raises the kinetic p*K*_a1_ by 0.36 units, whereas the orthogonal G352T mutation in YopH results in a full unit decrease.

The thermodynamic p*K*_a_ of the Cys nucleophile depends on the stabilization of the thiolate form provided by the neighboring histidine residue. Although no significant perturbation to this interaction was seen in our X-ray structures, or arose in the simulations, unobserved structural effects could be significant. For example, local protein unfolding in staphylococcal nuclease was shown to alter p*K*_a_ values and, conversely, affect the pH dependence of local protein fluctuations.^50^ It was important to determine whether unseen long-range structural effects of the mutation might affect the thermodynamic p*K*_a_ of the active-site Cys residue. This value was measured using the pH dependency of the rate of inactivation of the enzymes by iodoacetate, a method that has been applied previously in a number of proteins, including the PTPs YopH and VHR.^7,35^ The thermodynamic p*K*_a_ of the native YopH has been reported as 4.67 ± 0.15.^7^ Using conditions described in the Materials and Methods, we obtained the pH dependency for inactivation plotted in Figure S4, which yielded a value for the G352T of 4.3 ± 0.2 and 4.6 ± 0.2 for native YopH. Thus, the different pH dependency of the G352T variant arises not from substantial alteration of the thermodynamic p*K*_a_ of the thiol group of the cysteine side chain but predominantly from altered dynamic properties that affect the factors described in the Introduction, which distort the kinetic p*K*_a_ values from their thermodynamic values and affect the pH dependency of catalysis.

The crystal structures showing both ligand-free mutants crystallize in the loop-closed conformation suggest these mutations result in a shifted equilibrium to favor their closed-loop conformations. The crystal structures do not reveal obvious changes from the mutations that would rationalize a shift in equilibrium between the open and closed loops. Computational modeling was therefore used to assess how these mutations affect the respective populations of WPD-loop-open and -closed conformations.

### Computational Investigations into the Origin of the Implied Population Shifts

Our structural and kinetic analysis presented above demonstrate that the point mutations T177G in PTP1B and YopH G352T can alter the relative populations of the closed and open states of the WPD-loop. In order to help unravel the molecular mechanism(s) behind these population shifts, we performed MD simulations on both WT PTP1B and WT YopH and their respective point variants (T177G PTP1B and G352T YopH). For all four systems, we performed 10 replicas of 100 ns long MD simulations starting from the both the closed and open states (2 μs per system in total). The use of multiple replicas (like the 10 performed here) is important for obtaining both reliable and reproducible MD simulation data.^51^ We note that both we^18,52^ and others^53,54^ performed previous detailed computational studies of loop dynamics (or other large-scale conformational changes) using advanced enhanced sampling approaches that are able to (at least to some extent) quantitatively evaluate the populations observed between the different conformational states. This includes recent computational studies of WPD-loop dynamics in wild-type PTP1B and YopH.^18^ However, such studies are far from computationally trivial. In addition, while relevant NMR data exist that quantify the population difference between the two states in the wild-type enzymes,^17^ these differences are both quite subtle (in the range of 1–2 kcal mol^–1^ depending on whether a ligand is bound or not, making them hard to quantify computationally), and there also do not exist analogous data for the substituted variants studied in this work. However, as discussed below, our conventional MD simulations appear to be able to capture the population shift between different conformational states upon amino acid substitution and key qualitative features associated with this shift.

**Figure 6:**
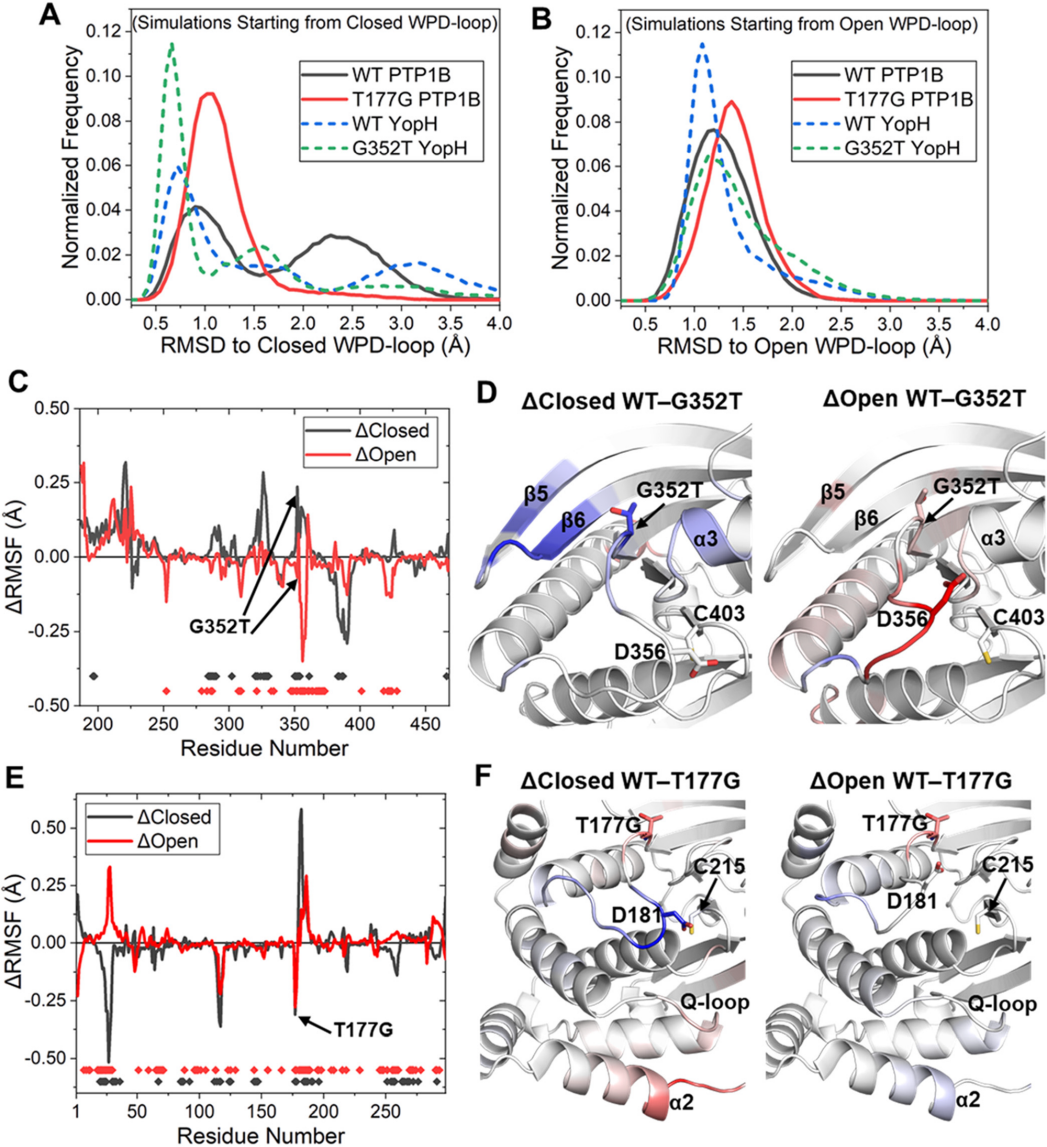
(A,B) Histograms of the C_α_ RMSD of the WPD-loop relative to its starting (crystal structure) position, obtained from 10 × 100 ns MD simulations starting from either the (A) closed or (B) open WPD-loop conformations. (C) Differences in calculated per residue C_α_ root mean square fluctuation (ΔRMSF) for the closed and open states between the WT and G352T variant of YopH, with a more positive value indicating increased flexibility in the WT residue. All simulations were performed on the ligand-free form of the enzyme. The small dots at the bottom of the graph represent ΔRMSF values that are identified as statistically significant as determined by a two-sample *t*-test (*p* < 0.05). Significant ΔRMSF differences for closed and open states are marked as black and red dots, respectively. (D) Color mapping of the significantly different (as determined by the above-described *t*-test) ΔRMSF values onto the structure of YopH. Color mapping is performed from blue (positive ΔRMSF) through to white (0 ΔRMSF value or not determined to be significantly different) to red (negative ΔRMSF). In practice, a blue residue would mean increased rigidity for the G352T variant over the WT, whereas a red residue would indicate increased flexibility of the G352T variant. (E,F) Same set of data as described for YopH in panels C and D but now for PTP1B instead (and the mutation T177G instead of G352T).

To assess the overall stability of the closed and open WPD-loop conformations for each system, we measured the C_α_ RMSD of the WPD-loop over the course of each simulation relative to its starting structure (i.e., X-ray structure with a closed or open WPD-loop). Focusing first on the closed state simulations (Figure 6A), we found that both point variants have a higher occupancy of sampling low RMSD states (i.e., those representing a closed WPD-loop) compared to that of their counterpart WT PTPs. These results are consistent with our X-ray data, in which, for both PTP1B and YopH, the ligand-free form is observed with a closed WPD-loop conformation (Figure 3). Interestingly, we also observe a (subtle) reduction in the stability of the open WPD-loop conformation for both point variants (Figure 6B), as their histograms are broader and generally sample greater RMSD values than their counterpart WT PTPs. This would therefore suggest that stabilization of both the closed state and destabilization of the open state are responsible for driving the experimentally observed population shifts toward the closed state for both point variants.

Our RMSD data presented above reflect differences in the overall stability of the WPD-loop, but in order to localize this effect to the per-residue level, we determined how the flexibility of the enzymes differed for both the closed and open states of the WPD-loop for the WT PTP against its counterpart point variant (Figure 6C–F). Given that our RMSD analysis above showed significant sampling of WPD-loop conformational states different from the starting structures, we only took forward MD simulation snapshots within 2 Å of the reference (crystal structure WPD-loop closed or open) state for analysis. Further, in order to ensure the differences identified were statistically significant, we performed a two-sample *t*-test (*p* < 0.05 used as cutoff) using the 10 replicas performed per system.

Focusing first on the differences in flexibility between the closed states of WT and G352T YopH (Figure 6C,D), we identified the G352T substitution to significantly rigidify the N-terminal portion of the WPD-loop where the substitution is located, alongside the nearby β5 and β6 strands and the α3-helix. For the open WPD-loop conformation, our MD simulations (Figure 6C,D) show that the G352T mutation has increased the flexibility of both the central and C-terminal portions of the WPD-loop alongside the α4-helix (which is directly connected to the C-terminal portion of the WPD-loop). These results are therefore consistent with our RMSD data (Figure 6A,B), in which both the closed state stabilization (including nearby secondary structure elements) and open state destabilization are responsible for the experimentally observed population shift toward the closed state for G352T YopH.

For the differences in flexibility between WT PTP1B and its T177G point variant (Figure 6E,F), we observe the point variant to be directly destabilizing toward both its own position on the loop and that of the neighboring T188 residue in both the open and closed states of the WPD-loop. Given the nature of the mutation (threonine to the less conformationally constrained glycine) and resulting loss of side chain interactions, these results are unsurprising. Interestingly, however, this mutation has an indirect (and compensatory) effect of rigidifying parts of the central and C-terminal portions for both the WPD-loop’s closed and open states, with the magnitude of this rigidification being notably stronger for the closed state of the WPD-loop (Figure 6E,F). Taken together with our RMSD analysis (Figure 6A,B), this compensatory effect appears to result in an overall more rigid WPD-loop for T177G PTP1B when in the closed state, in contrast to a more flexible WPD-loop open state for T177G PTP1B (when compared to WT PTP1B).

Interestingly, we also observe the T177G mutation to alter the stability of residues around the Q-loop and α2- and α5-helices. These regions have recently been implicated to be part of the PTP1B allosteric network through a combined mutagenesis and bioinformatics study.^55^ The sensitivity of this region to the T177G point mutation observed in our MD simulations for both the closed and open states adds further weight to this argument.

### Changes in the Local Hydrogen Bonding Network Help Rationalize the Observed Population Shifts

**Figure 7:**
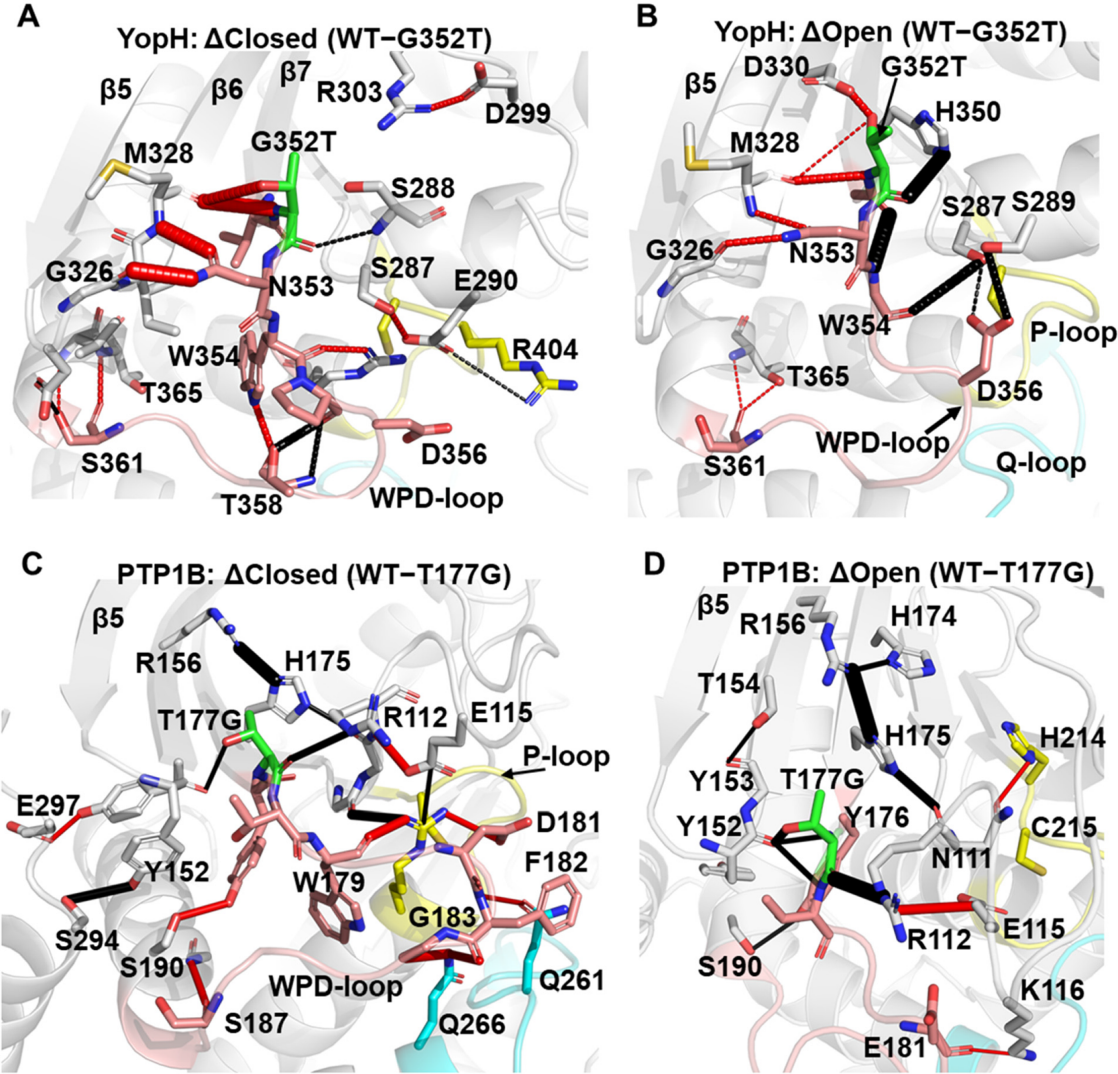
(A,B) Differences between the hydrogen bonding (H-bonding) interaction networks formed for the WT and G352T point variant of YopH in the WPD-loop (A) closed and (B) open conformations. H-bonding interactions which have a higher occupancy in the WT are shown as black dashes between the donor and acceptor atom, with red dashes used to indicate H-bonding interactions which have a higher occupancy in the G352T variant. The width of the dash indicates the magnitude of the difference in the occupancy of the hydrogen bond between the two enzymes. (C,D) These panels show the same data as shown for YopH in panels A and B but now illustrate instead the differences between WT and T177G PTP1B in their WPD-loop (C) closed and (D) open WPD-loop states, respectively.

Our RMSD and RMSF calculations described above helped identify how both point variants altered the stability of both the open and closed states of the WPD-loop relative to their counterpart WT PTPs. To understand how these structural effects manifest, we analyzed the changes in the hydrogen bonding (H-bonding) interaction networks between both WT PTPs and their respective point variants for both the closed and open states (Figure 7). We note that while we utilized multiple replica simulations to reduce the risk of noise based differences in H-bonding interactions, we further limited our search to differences in H-bonding interactions close to the mutation site or on the WPD-loop itself and, further, only included those interactions with differences of at least 15% greater occupancy in one state over the other (see the Materials and Methods for further details).

For YopH and the G352T variant in the WPD-loop closed state (Figure 7A), we observe the G352T variant to form higher occupancy interactions with the backbone carbonyl of M328 on the β6-strand (both through the main chain nitrogen and the side chain hydroxyl group only present in the point variant). Further, the neighboring N353 side chain forms notably higher occupancy H-bonding interactions with the backbones of M328 and G326 than in the WT simulations. These higher occupancy interactions help explain the increased rigidity observed in the loop closed state for both the WPD-loop and surrounding regions (Figure 6C,D).

For YopH in the open state (Figure 7B), while many of the same interactions as described for the closed state are stronger (but to a lesser extent) in the point variant, we also observe the WT to make many higher occupancy hydrogen bonds. For example, G352 makes significantly higher occupancy interactions with H350 and W354, which would suggest the additional conformational restriction placed on the backbone dihedrals (in the point variant G352T) prevents/reduces the sampling of these stabilizing interactions in the open state.

Focusing for now on the differences between WT PTP1B in both the open and closed WPD-loop states, the direct impact of the T177G mutation is clearly detrimental toward its own interactions with Y152 and R112, with reduced occupancy of hydrogen bonding interactions compared to what is observed in the WT enzyme (Figure 7C,D). The T177A substitution was previously identified by Cui et al.^49^ to also induce a population shift through destabilization of the open state (by disrupting an interaction network between T178, T177, and H175). Here, we show an extension of the disruption of this network following from H175 to R156 of the β6-strand and N111 and E112 on the E-loop (Figure 7C,D). We note that while this same network is disturbed in both the open and closed states upon mutation (which is consistent with the immediate site surrounding the WPD-loop becoming more unstable as seen in our RMSF calculations, Figure 6E,F), the effect appears to be stronger in the open state, especially when considering R112’s H-bonding interaction with the backbone of T177G. Further, for the closed state of PTP1B, we observed differences in the hydrogen bonding patterns of the closed WPD-loop conformation between residues on the central portion of the WPD-loop (W179, D181, F182, G183) and the neighboring Q- and P-loops (Figure 7C), likely explaining the increased rigidity for this region seen in our RMSF calculations (Figure 6E,F).

## Conclusions

Catalysis in PTPs relies on the closure of a flexible protein loop carrying a residue acting as a general acid in the first step of the mechanism and as a base in the second step. The orthogonal substitutions of a single, noncatalytic residue in the WPD-loops of YopH and PTP1B result in no loss of maximal catalytic activity but in shifted pH-rate profiles exhibiting an altered kinetic p*K*_a_ of the nucleophilic cysteine residue. Surprisingly, the G352T variant of YopH has no loss of activity at optimal pH but activity at low pH significantly higher than that of native YopH, reflected in a broadened pH-rate profile on the acid side. Fitting the pH-rate data reveals a significantly lower apparent, or kinetic, p*K*_a_ of the nucleophilic C402. Titration of this residue showed a very modest change in the thermodynamic p*K*_a_, meaning the altered kinetic p*K*_a_ results from changes in other processes affecting the catalytic mechanism. Changes in the corresponding PTP1B T177G variant are more modest and in the opposite direction; the pH profile is slightly narrowed, and the variant is less active in the most acidic range of the profile. Crystal structures show no structural perturbations result from the substitutions but imply the WPD-loop closed conformation has become more favored in the ligand-free enzyme, in contrast to the native enzyme.

Our computational analysis further supports the conclusion that both variants have a higher occupancy of the loop-closed conformation than their WT counterparts and shows that the shift results from a combination of increased stability of the closed state of the WPD-loop, with concomitant destabilization of the corresponding loop-open state. Simulations identified the origins of this population shift and also revealed unexpected differences in the flexibility of the WPD-loop and neighboring regions that result from these mutations, showing these single point mutations can substantially disrupt the conformational dynamics of many residues in the active site and neighboring regions. Specifically, relative to the native enzyme, the G352T substitution in YopH makes the N-terminal portion of its WPD-loop and nearby secondary structural elements more rigid in the loop-closed state, while simultaneously increasing the flexibility of the central and C-terminal portions of the loop in the open state. In the case of PTP1B, the T177G substitution results in an obvious loss of hydrogen bonding interactions at the site of substitution in both the closed and open states. However, when combined with changes to the remainder of the WPD-loop, it results in a more rigid closed loop and a more flexible open loop.

The involvement of nonchemical processes in perturbing the observed p*K*_a_ values of ionizable catalytic residues obtained from enzyme kinetics studies has been well-documented.^21−23^

While this can be a bane to enzymologists, it affords nature a tool to evolve the pH dependency of an enzyme by a more facile and less disruptive manner than altering the electrostatic network around catalytic residues themselves or changing them. Protein motions are one such process that, as the results shown here demonstrate, can be altered in a manner such that a significant shift in the pH dependence of activity can result. Although an area of growing study, the role of protein dynamics in phosphatases is less well-known than in their catalytic counterparts, kinases, where significant roles of protein motions are well-documented.^56,57^ Aside from three highly conserved residues, PTPs have significant sequence variation within their WPD-loops (Figure S1), the mobility of which is critical for catalysis. Those differences, and the altered dynamics of loop motion that result, likely contribute to the variation in pH dependency seen among the PTP family despite their shared catalytic residues and mechanism. The results presented here mean that evaluating the effect of mutations in PTPs, or other enzymes, by comparing the activity of variants with the WT enzyme at a single pH may not give a complete picture of the actual impact of the amino acid substitutions. More generally, these results show a previously unappreciated means by which nature can alter the pH dependency of catalysis, by employing a phenomenon that bedevils the interpretation of pH-rate profiles by experimentalists.

## Supporting Information Available

Additional simulation methodology details and supplemental structural analysis (PDF)
