# Supplementary material for: Single Residue on the WPD-Loop Affects the pH Dependency of Catalysis in Protein Tyrosine Phosphatases
Source: JACS Au. 2021 Apr 23;1(5):646–59. doi: 10.1021/jacsau.1c00054 (PMC8297725; doi:10.1021/jacsau.1c00054)
Supplement: Supplementary file 1 — au1c00054_si_001.pdf [file au1c00054_si_001.pdf]

Supporting Information:

Single Residue on the WPD-Loop Affects the pH Dependency of Catalysis in Protein Tyrosine Phosphatases

Ruidan Shen,[‡] Rory M. Crean,[†] Sean J. Johnson,[*,‡] Shina C. L. Kamerlin[*,†], and Alvan C. Hengge,[*,‡]

[‡] Department of Chemistry and Biochemistry, Utah State University, Logan, Utah 84322-0300, United States.

[†] Science for Life Laboratory, Department of Chemistry − BMC, Uppsala University, BMC, Box 576, S-751 23 Uppsala, Sweden.

Table of Contents

Supplementary Methodology S2

Equilibration Procedure for Conventional Molecular Dynamics Simulations *S2*

Supplementary Figures S4

Supplementary Tables S10

Supplementary References S13

Supplementary Methodology

Equilibration Procedure for Conventional Molecular Dynamics Simulations

The following procedure was used for all systems in order to prepare for production MD simulations. All simulations were run using an 8 Å direct space non-bonded cut-off, with long range electrostatics evaluated using the particle-mesh Ewald[1] method. Further, all dynamics step had the SHAKE[2] algorithm applied to constrain all bonds containing a hydrogen atom. MD simulations were performed using Amber 18.[3]

All hydrogen atoms and solvent molecules (including any counterions) were energy minimized using first 500 steps of steepest descent minimization, followed by 500 steps of conjugate gradient minimization. In order to keep the protein and substrate fixed in place during the minimization, 10 kcal mol^{-1} $Å^{-2}$ positional restraints were applied to all heavy (non-hydrogen) protein and ligand atoms. These positional restraints were retained as the system was heated from 50 to 300 K in an NVT ensemble over the course of 200 ps of simulation time using a 1 fs time step and Langevin temperature control[4] (collision frequency of 1 ps^{-1}). For the subsequent steps, the restraints were reduced from 10 to 5 kcal mol^{-1} $Å^{-2}$ and applied to only the C_α atoms of all protein residues. Following this, another energy minimization and heating process was performed, again using 500

steps of steepest descent minimization followed by 500 steps of conjugate gradient minimization. The restraints on the C_α atoms were retained as the system was heated from 25 K to 300 K over the course of 500 ps in an NVT ensemble using the same conditions as described above. Simulations were then performed in the NPT ensemble (300 K, 1 atm) using Langevin temperature control[4] (collision frequency of 1 ps^{-1}) and a Berendsen barostat[5] (1 ps pressure relaxation time). NPT simulations were performed using a 2 fs simulation time step. The 5 kcal mol^{-1} $Å^{-2}$ positional restraints described above were slowly released in 1 kcal mol^{-1} $Å^{-2}$ increments every 10 ps of 50 ps of simulation time. Finally, a 1 ns long unrestrained MD simulation was performed for further equilibration, using the same NPT conditions as described above, before performing the final 100ns production runs (10 replicas per system, for equilibration of the production runs see **Figures S5** and **S6**).

Supplementary Figures

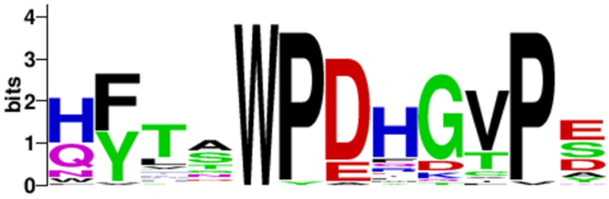

Figure S1. Sequence logo of the WPD-loop based on a library of classical active PTPs, as defined by Chen *et al.*[6], as well as the canonical YopH sequence. The multiple sequence alignment (MSA) was generated using the MUSCLE webserver utility[7], and gappy positions (>50%) were stripped. The resulting MSA was converted into a sequence logo using Weblogo3[8], only visualizing the 12 WPD loop residues.

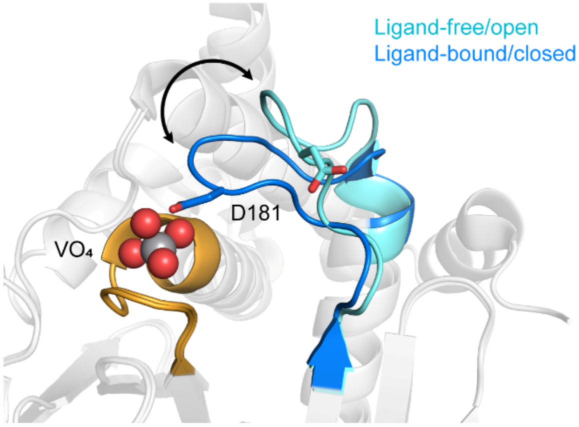

Figure S2. Overlay of the different conformations of the WPD loop of PTP1B. Shown here are the structures of the open (blue, PDB ID: 2CM2[9]) and closed (gold, PDB ID: 3I80[10]) forms of the WPD loop (the closed conformation contains a vanadate ion in the active site). As can be seen, the remainder of the scaffold remains virtually identical between the two proteins. This conformational change moves the conserved Asp residue (D181, PTP1B numbering) ~9Å, in order to optimally position it for catalysis. The ligand-free WPD-loops are colored in cyan, ligand-bound WPD-loops are colored in blue, and P-loops are colored in yellow.

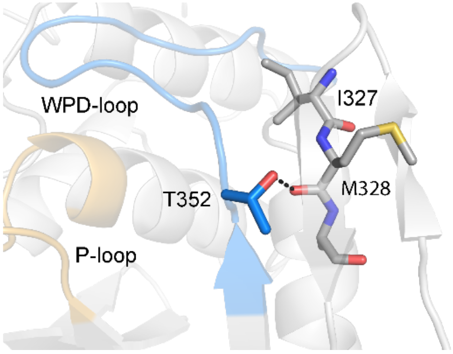

Figure S3. Substitution of G352 to Thr in YopH introduces an additional hydrogen bond between the side chain of T352 and backbone carbonyl of M328, that is not present in the wild-type enzyme.

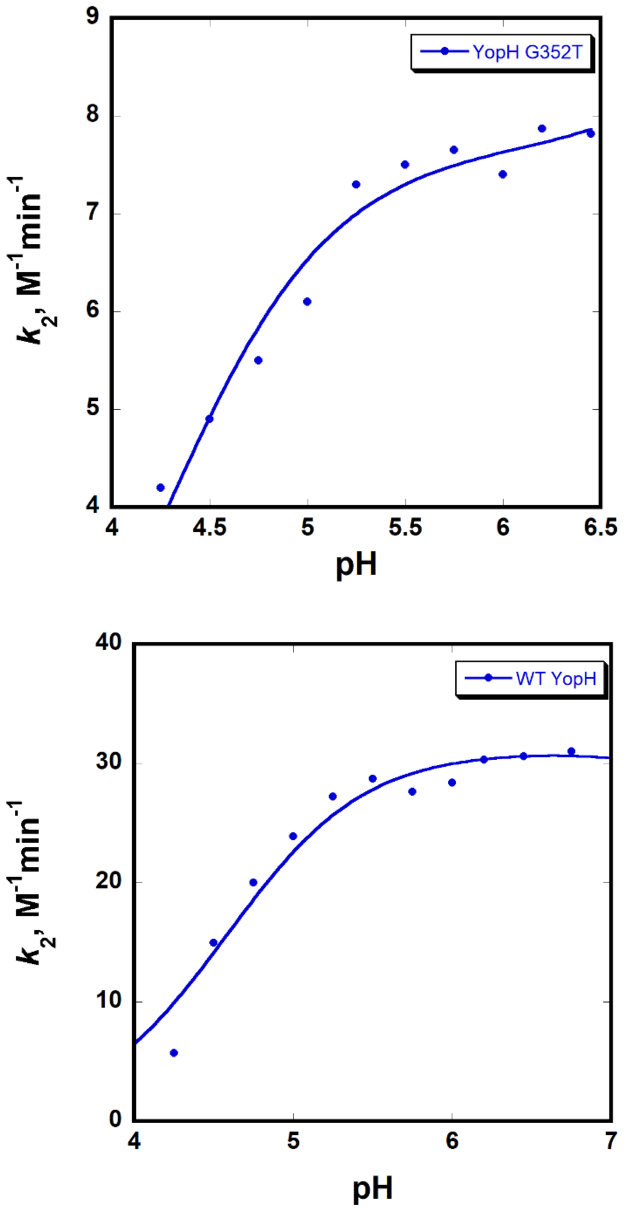

Figure S4. Active site cysteine thiol titration curves were constructed by plotting the second-order rate constant for inactivation by iodoacetate of WT and G352T YopH against pH (4.25-6.75). The thermodynamic pK_a of Cys215 is determined from fitting the half-bell pH-inactivation profile to Eq. 2 of the main text.

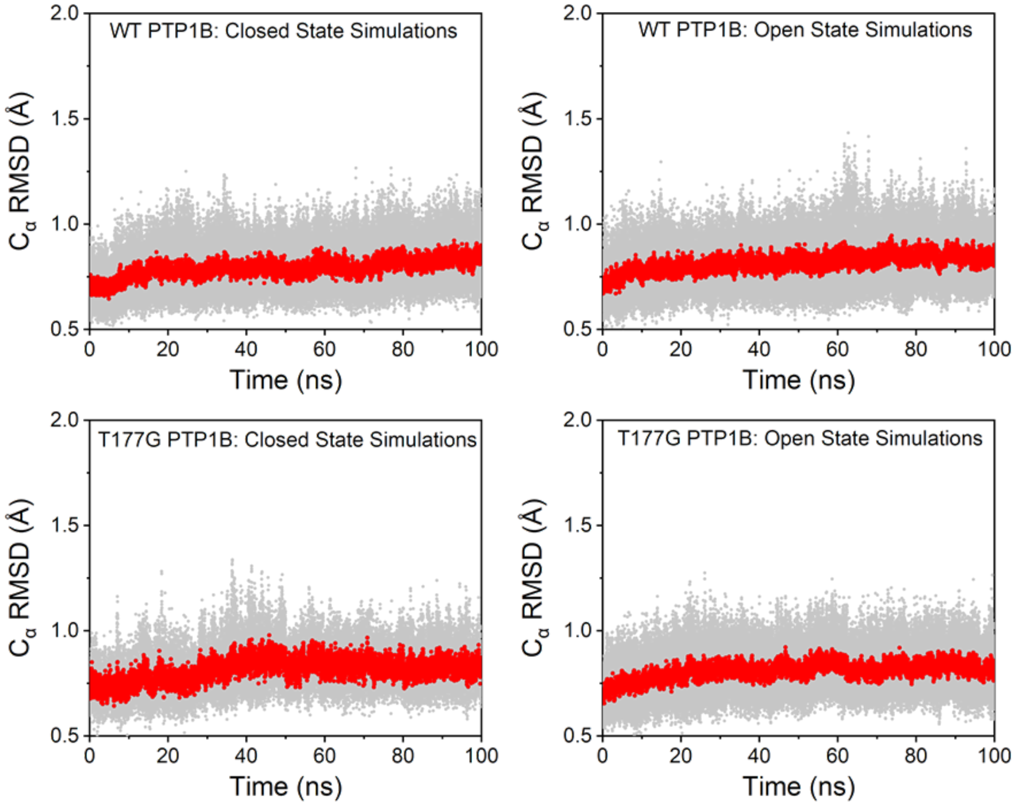

Figure S5. Root mean square deviations (RMSD, Å) of all backbone C_α atoms over the course of our MD simulations (10 x 100 ns for each system, see the **Methodology** section for further details) for (**top**) WT and (**bottom**) T177G PTP1B. The C_α RMSD is measured to the whole enzyme (*i.e.* every residue) using the appropriate crystal structure as the reference conformation. All simulations initiated starting from the closed WPD-loop conformation used a closed crystal structure as reference (PDBs: 6B90[11] and 7L0C [this study] for WT and T177G PTP1B respectively), whilst simulations starting from the open WPD-loop conformation used an open crystal structure as reference (PDB: 6B90[11] for both WT and T177G PTP1B). The red dots denote the rolling average from the 10 replicas, while the grey dots show the results from each individual run. In all cases, PTP1B residues that make up the "recognition domain" (residues 28-56) were not included in the RMSD calculation, due to their high flexibility (this region has very limited secondary structure).

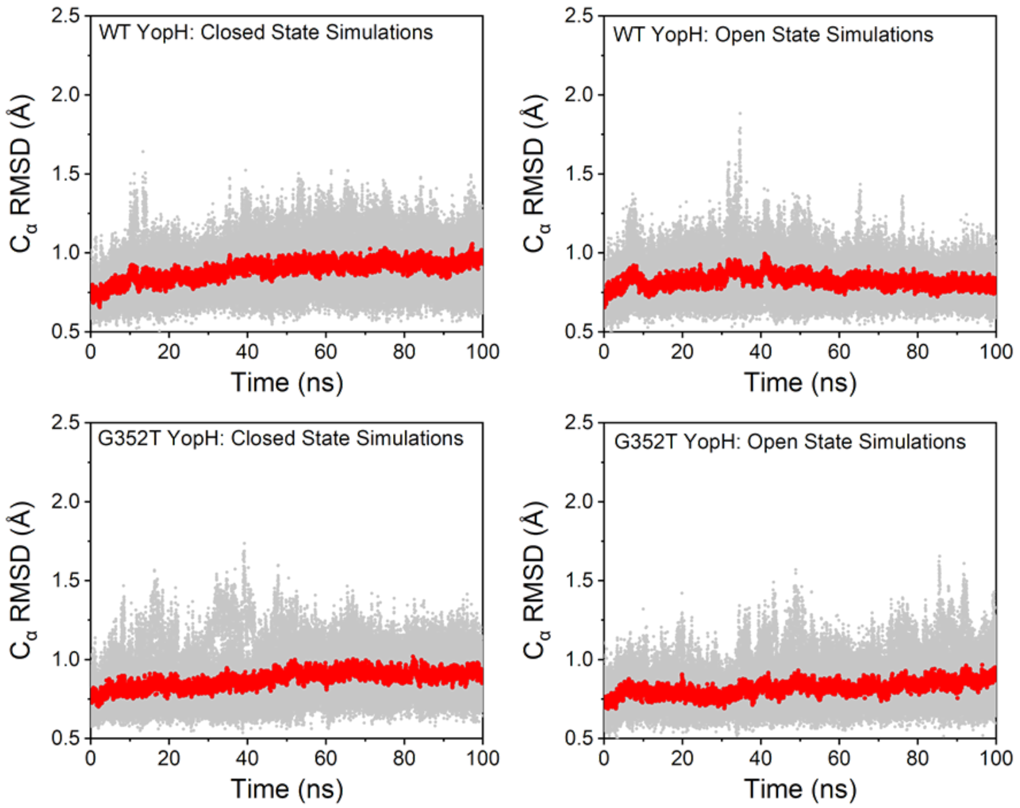

Figure S6. Root mean square deviations (RMSD, Å) of all backbone C_α atoms over the course of our MD simulations (10 x 100 ns for each system, see the **Methodology** section for further details) for (**top**) WT and (**bottom**) G325T YopH. The C_α RMSD is measured to the whole enzyme (*i.e.* every residue) using the appropriate crystal structure as the reference conformation. All simulations initiated starting from the closed WPD-loop conformation used a closed crystal structure as reference, (PDBs: 2I42[12] and 7L0I [this study] for WT and G352T YopH respectively), whilst simulations starting from the open WPD-loop conformation used an open crystal structure as reference (PDB: 1YPT[13] for both WT and G352T YopH). The red dots denote the rolling average from the 10 replicas, while the grey dots show the results from each individual run. In all cases, PTP1B residues that make up the "recognition domain" (residues 48-84) were not included in the RMSD calculation, due to their high flexibility (this region has very limited secondary structure).

Supplementary Tables

Table S1. WPD-loop sequences for WT PTP1B, PTP1B T177G, YopH G352T and WT YopH.[a]

System	Sequence
PTP1B	$_{175}$HYTTWPDFGVPES$_{187}$
PTP1B T177G	HYGTWPDFGVPES
YopH G352T	HVTNWPDQTAVSS
YopH	$_{350}$HVGNWPDQTAVSS$_{362}$

[a] The WPD-loop sequence for WT PTP1B is colored in red, while that for WT YopH is colored in blue.

Table S2. The forward and reverse primers used to construct PTP1B T177G and YopH G352T.

PTP1B T177G Forward Primer	5'-TTTCCACTATGGCACATGGCCTG-3'
PTP1B T177G Reverse Primer	5'-TGTAAGATCTCTCGAGTTTC-3'
YopH G352T Forward Primer	5'-GGTTCATGTTACCAATTGGCCC-3'
YopH G352T Reverse Primer	5'-ACAGGAACAGAAATTGTTTTTG-3'

Table S3. All X-ray crystal structures used for our MD simulations of PTP1B and YopH in their ligand-free forms.

PTP and WPD-loop conformation	Crystal Structure Used	Modifications/Mutations Required
**WT PTP1B**		
Closed WPD-loop[a]	6B90[11]	None.
Open WPD-loop[a]	6B90[11]	None.
**WT YopH**		
Closed WPD-loop	2I42[12]	Bound VO_4 ion removed.
Open WPD-loop	1YPT[13]	No.
**T177G PTP1B**		
Closed WPD-loop	7L0C[b]	No.
Open WPD-loop	6B90[11]	T177G substitution made with PyMol
**G352T YopH**		
Closed WPD-loop	7L0I[b]	No.
Open WPD-loop	1YPT[13]	G352T substitution made with PyMol

[a] Structure contains both closed and open conformations of the WPD-loop. [b] Structures used were generated from this study.

Table S4. Non-standard protonation states and histidine tautomerization states used in all simulations.[a]

System	Non-Standard Protonation States	HIE Tautomerization State[b]
**WT/T177G PTP1B**	D181 – Deprotonated<br>C215 – Deprotonated<br>E102 – Protonated	25, 54, 60, 94, 173, 175, 208, 296
**WT/G352T YopH**	D356 – Deprotonated<br>C403 – Deprotonated	270, 350

[a] The same protonation and tautomerization states were used for both the WT and its corresponding point variant.

[b] HIE corresponds to a neutral histidine residue which is protonated on its $N_{\epsilon 2}$ nitrogen atom. All other histidine residues were simulated as neutral and protonated on their $N_{\delta 1}$ nitrogen (HID).

Supplementary References

1. Darden, T.; York, D.; Pedersen, L., Particle Mesh Ewald: An N·log(N) Method for Ewald Sums in Large Systems. *J. Chem. Phys.* **1993,** *98*, 10089-10092.

2. Ryckaert, J.-P.; Ciccotti, G.; Berendsen, H. J. C., Numerical Integration of the Cartesian Equations of Motion of a System with Constraints: Molecular Dynamics of N-Alkanes. *J. Comput. Phys.* **1977,** *23*, 327-341.

3. Case, D. A.; Ben-Shalom, I. Y.; Brozell, S. R.; Cerutti, D. S.; Cheatham III, T. E.; Cruzeiro, V. W. D.; Darden, T. A.; Duke, R. E.; Ghoreishi, D.; Gilson, M. K.; Gohlke, H.; Goetz, A. W.; Greene, D.; Harris, R.; Homeyer, N.; Izadi, S.; Kovalenko, A.; Kurtzman, T.; Lee, T. S.; LeGrand, S.; Li, P.; Lin, C.; Liu, J.; Luchko, T.; Luo, R.; Mermelstein, D. J.; Merz, K. M.; Miao, Y.; Monard, G.; Nguyen, C.; Nguyen, H.; Omelyan, I.; Onufriev, A.; Pan, F.; Qi, R.; Roe, D. R.; Roitberg, A.; Sagui, C.; Schott-Verdugo, S.; Shen, J.; Simmerling, C. L.; Smith, J.; Salomon-Ferrer, R.; Swails, J.; Walker, R. C.; Wang, J.; Wei, H.; Wolf R.M.; Wu, X.; Xiao, L.; York, D. M.; Kollman, P. A. *AMBER 2018*, University of California: San Francisco, 2018.

4. Schneider, T.; Stoll, E., Molecular-Dynamics Study of a Three-Dimensional One-Component Model for Distortive Phase Transitions. *Phys. Rev. B* **1978,** *17*, 1302-1322.

5. Berendsen, H. J. C.; Postma, J. P. M.; van Gunsteren, W. F.; DiNola, A.; Haak, J. R., Molecular dynamics with coupling to an external bath. *J. Chem. Phys.* **1984,** *81*, 3684-3690.

6. Chen, M. J.; Dixon, J. E.; Manning, G., Genomics and Evolution of Protein Phosphatases. *Sci. Signal.* **2017,** *10*.

7. Madeira, F.; Park, Y. M.; Lee, J.; Buso, N.; Gur, T.; Madhusoodanan, N.; Basutkar, P.; Tivey, A. R. N.; Potter, S. C.; Finn, R. D.; Lopez, R., The EMBL-EBI Search and Sequence Analysis Tools APIs in 2019. *Nucleic Acids Res.* **2019,** *47*, W636-W641.

8. Crooks, G. E.; Hon, G.; Chandonia, J. M.; Brenner, S. E., WebLogo: A Sequence Logo Generator. *Genome Res.* **2004,** *14*, 1188-1190.

9. Ala, P. J.; Gonneville, L.; Hillman, M. C.; Becker-Pasha, M.; Wei, M.; Reid, B. G.; Klabe, R.; Yue, E. W.; Wayland, B.; Douty, B.; Polam, P.; Wasserman, Z.; Bower, M.; Combs, A. P.; Burn, T. C.; Hollis, G. F.; Wynn, R., Structural Basis for Inhibition of Protein-Tyrosine Phosphatase 1B by Isothiazolidinone Heterocyclic Phosphonate Mimetics. *J. Biol. Chem.* **2006,** *281*, 32784-32795.

10. Brandao, T. A.; Hengge, A. C.; Johnson, S. J., Insights into the Reaction of Protein-Tyrosine Phosphatase 1B: Crystal Structures for Transition State Analogs of Both Catalytic Steps. *J. Biol. Chem.* **2010,** *285*, 15874-83.

11. Keedy, D. A.; Hill, Z. B.; Biel, J. T.; Kang, E.; Rettenmaier, T. J.; Brandão-Neto, J.; Pearce, N. M.; von Delft, F.; Wells, J. A.; Fraser, J. S., An Expanded Allosteric Network in PTP1B by Multitemperature Crystallography, Fragment Screening, and Covalent Tethering. *eLife* **2018,** *7*, 1-36.

12. Denu, J. M.; Lohse, D. L.; Vijayalakshmi, J.; Saper, M. A.; Dixon, J. E., Visualization of Intermediate and Transition-State Structures in Protein-Tyrosine Phosphatase Catalysis. *Proc. Natl. Acad. Sci. U. S. A.* **1996,** *93*, 2493-2498.

13. Stuckey, J. A.; Schubert, H. L.; Fauman, E. B.; Zhang, Z.-Y.; Dixon, J. E.; Saper, M. A., Crystal structure of Yersinia Protein Tyrosine Phosphatase at 2.5 Å and the Complex with Tungstate. *Nature* **1994,** *370*, 571-575.
